# Identification and functional validation of a novel pathogenic *POT1* germline variant p.G95V in familial melanoma

**DOI:** 10.1002/jvc2.382

**Published:** 2024-02-13

**Authors:** Farrah Bakr, Anjana Kulkarni, Stephen Mounsey, Tracey Mitchell, Sean Whittaker, Katie Lacy

**Affiliations:** ^1^ St John's Institute of Dermatology Guy's and St Thomas' NHS Trust London UK; ^2^ Department of Clinical Genetics Guy's and St Thomas’ NHS Trust London UK

**Keywords:** familial melanoma, germline mutation, germline variant, melanoma, melanoma‐susceptibility genes, POT1, telomere maintenance

## Abstract

*POT1* variants have been identified in familial melanoma (FM) as well as a number of other germline and somatic malignancies. The functional validation of variants identified from the screening of patients with melanoma gene susceptibility panels is key to understanding the clinical significance of identified variants. Here we report a novel, likely pathogenic *POT1* missense variant (p.G95V) in FM and investigate its functional impact. We demonstrate loss of function owing to the inability of the mutant POT1 protein to bind telomeric DNA compared to its wild‐type counterpart. This study provides important functional validation of a novel *POT1* variant in FM.

## Graphical abstract

*POT1* variants have been identified in familial melanoma as well as a number of other germline and somatic malignancies. Here we report a novel, likely pathogenic *POT1* missense variant (p.G95V) in familial melanoma and investigate its functional impact. We demonstrate loss of function owing to the inability of the mutant POT1 protein to bind telomeric DNA compared to its wild‐type counterpart. This study provides important functional validation of a novel *POT1* variant in familial melanoma.

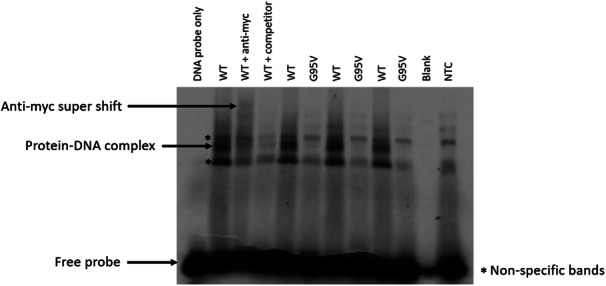

## INTRODUCTION

Familial melanoma (FM) is an inherited form of the diease in which at least two first‐degree relatives are diagnosed with melanoma. Occurring in 1−10% of all melanoma patients, it is associated with several high‐penetrance susceptibility genes, most commonly cyclin‐dependent kinase inhibitor 2 A (*CDKN2A*) in 20−40% of FM cases.^1^ Less commonly *CDK4* and *BAP1* have also been identified^2^ and recently, genes involved in telomere maintenance: *POT1, ACD* and *TERF2IP*,^3^ but over 50% of suspected FM cases have no identifiable germline mutation. Telomere instabilility is a key feature of melanoma given the high frequency of somatic TERT promoter variants identified in sporadic melanoma, however, TERT germline variants are only detected rarely in FM.^4^

Protection of Telomeres 1 (POT1) is a component of the Shelterin complex (POT1, TRF1, TRF2, TIN2, TPP1 and RAP1) which binds to the ends of telomeres, critical for telomere end protection. POT1 specifically binds to the repetitive sequence of single stranded DNA at the very end of the telomere (5′TTAGGG3′) via its N‐terminal oligonucleotide binding (OB) domains. As such, POT1 plays a vital role in maintaining chromosomal stability by preventing aberrant DNA damage pathway activation at telomeres and regulating telomerase activity.

There is mounting evidence that loss of function *POT1* variants are implicated in malignancy. *POT1* germline variants have been identified in a number of familial cancer syndromes most commonly melanoma, chronic lymphocytic leukaemia, and familial cardiac angiosarcoma.^3^, ^5^, ^6^, ^7^ *POT1* germline variants have been found in 2−4% of CDKN2A/CDK4‐negative FM pedigrees, indicating that *POT1* is the second major melanoma susceptibility gene following CDKN2A.^8^ *CDK4* mutations have been reported with a prevalence of 0.68%.^2^ *POT1* mutations are rare and contribute to a 0.5% melanoma risk burden in the general population. However, functional validation of variants is critical in light of increased melanoma genetic screening in the clinic. *POT1* germline variants have also been identified in familial glioma, including p.G65C, which occurs at the same amino acid residue as the one reported in this study but has not been functionally validated to date. Germline mutations have also been reported in papillary thyroid cancer, one of which (p.V29L) has been shown to have disrupted telomere binding.^6^, ^9^

## CASE REPORT

A 79 year‐old lady presented in 2008 with a history of 12 melanomas from age 32 and during subsequent surveillance she was diagnosed with five early stage melanomas (stage pT1a), nine in‐situ melanomas and seven basal cell carcinomas. She developed a papillary thyroid cancer (pT1N0M0), surgically excised in 2010.

The patient had a strong family history of malignancy. Her daughter had four melanomas from the age of 30 and died of gastric cancer age 45. Her son died aged 37 from a glioblastoma. In view of her personal and family history of multiple melanomas, she underwent germline genetic testing following counselling by the clinical genetics team with a melanoma susceptibility gene panel and a variant of uncertain significance in the *POT1* gene (c.284 G > T p.(Gly95Val)) was identified.

## DISCUSSION

**Figure 1:**
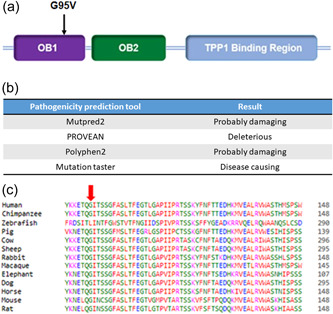
The POT1 p.G95V missense variant is predicted to be pathogenic and disrupts telomere binding. (a) Mutant localisation to the oligonucleotide binding domain (OB1) on a schematic of the protein. (b) p.G95V is predicted to have a deleterious effect on protein structure and/or function by four independent pathogenicity prediction tools (MutPred2, Provean, Polyphen2 and Mutation taster). (c) Multiple sequence alignment of the POT1 protein in humans and 12 other evolutionary diverse species. Amino acid positions 89−148, relative to human POT1, are shown with the arrow indicating the highly conserved p.G95V residue.

**Figure 2:**
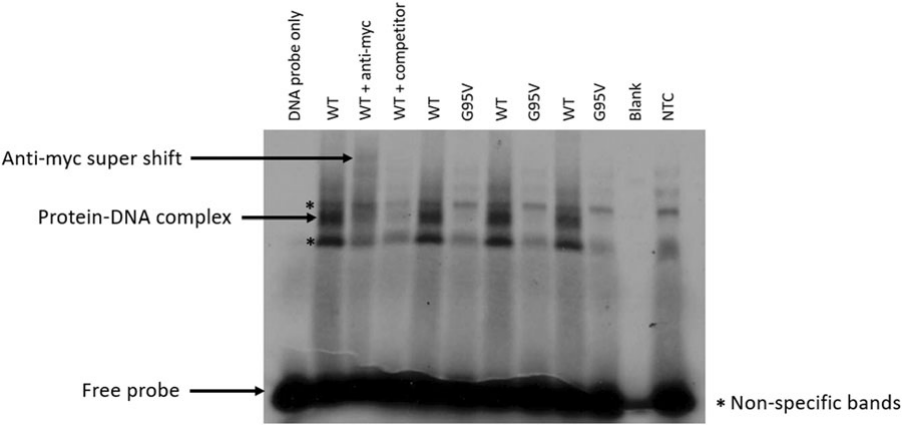
The POT1 p.G95V missense variant disrupts telomere binding. Electrophoretic mobility shift assay demonstrates the binding of in vitro translated wild‐type (WT) and mutant p.G95V POT1 protein to a γ‐p33‐labelled single‐stranded telomere oligonucleotide containing the POT1 target binding sequence. WT reaction shows formation of the POT1‐telomere complex. As the recombinant IVT POT1 proteins are myc‐tagged, 2.5 μg anti‐myc antibody (9E10) (Santa Cruz Biotechnology) was added to the EMSA reaction. The resulting supershift clearly demonstrates the specificity of the DNA‐WT protein interaction. The protein‐DNA complex is effectively competed out by an unlabelled telomere probe (competitor). Abolition of binding is observed in POT1 p.G95V protein. No band is seen in the no template control. Excess free probe demonstrates that the binding reaction is not limited by lack of available protein. Representative EMSA from three biological repeats. POT1, Protection of Telomeres 1.

There is strong evidence that this novel germline variant is likely to be a variant of significance. Firstly, the affected amino acid resides in the functionally critical OB1 domain (Figure 1a). Furthermore, it is predicted to be damaging by a consensus of five independent bioinformatic tools (Figure 1b) and is classified as pathogenic according to ACMG guidelines.^10^ The mutated amino acid residue is phylogenetically conserved which suggests it is critical to POT1 function (Figure 1c). In addition, the ability of in‐vitro translated POT1 p.G95V to bind to telomeric DNA was investigated using electrophoretic mobility shift assay (EMSA) (Figure 2), where in contrast to wild type protein, a complete lack of POT1‐DNA complex formation was observed.

For the first time, this study has functionally validated the p.G95V POT1 variant. We provide evidence that this is a loss of function variant owing to its inability to bind to telomeres, a finding consistent with other reported OB domain POT1 variants.^3^, ^7^, ^9^ Interestingly, an identical somatic POT1 variant has been identified in chronic lymphocytic leukemia, which further highlights its likely driver mutation status.^11^ Furthermore, a POT1 variant (p.G65C) which occurs at the same amino acid residue as the one reported in this study, has been identified in familial glioma and is also predicted to disrupt telomere binding.^6^

POT1 loss has been associated with activation of the Ataxia Telangiectasia and Rad3‐related protein (ATR) pathway.^12^ Given melanoma is a UV driven malignancy it is unsurprising that mutations in genes encoding DNA damage repair proteins have been reported in a subset of melanomas. In one study, mutations affecting the ATR pathway (*ATR, Chk1, 53BP1, RPA*) occur in 16−25% of cases and the ATM pathway (*ATM, MDC1, Chk2*) in 8−22% of cases.^13^ As ATR inhibitors are currently under evaluation in clinical trials for the management of a variety of solid tumours, targeting this pathway could be relevant in patients harbouring *POT1* variants.

In summary, the identification of a pathogenic *POT1* gene variant with demonstrated functional relevance, along with the advent of new potential therapeutic agents that target DNA damage repair pathways, could have wider implications for genetic screening and the management of other FM patients with *POT1* variants. In addition, this patient's family history suggests that POT1 loss of function germline variants can also predispose to a variety of other malignancies.

## AUTHOR CONTRIBUTIONS

Farrah Bakr designed and performed the experiments, analysed the data and wrote the manuscript. Anjana Kulkarni helped with interpretation of results from genetic analyses and review of the manuscript. Stephen Mounsey was involved in data collection and review of the manuscript. Tracey Mitchell and Sean Whittaker were involved with designing the study and review of the manuscript. Katie Lacy was involved with clinical management of the patient, design of the study and review of the manuscript.

## CONFLICTS OF INTEREST STATEMENT

The authors declare no conflict of interest.

## ETHICS STATEMENT

All patients in this manuscript have given written informed consent for participation in the study and the use of their deidentified, anonymized, aggregated data and their case details (including photographs) for publication. Ethical Approval: not applicable.

## Data Availability

Data sharing not applicable to this article as no datasets were generated or analysed during the current study.
